# A Brazilian case of IFAP syndrome with severe congenital ichthyosis and limb malformations caused by a rare variant in *MBTPS2*

**DOI:** 10.1590/1984-0462/2023/41/2022057

**Published:** 2023-04-07

**Authors:** Michele Patricia Migliavacca, Rodrigo Ambrosio Fock, Nadia Almeida, Thereza Cavalcanti, Darine Villela, Ana Beatriz Alvarez Perez, David Valle, Elizabeth Wohler, Nara Lygia de Macena Sobreira, Salmo Raskin

**Affiliations:** a Universidade Federal de São Paulo, São Paulo, SP, Brazil.; b Johns Hopkins University School of Medicine, Baltimore, MD, USA.; c Pontifícia Universidade Católica do Paraná, Curitiba, PR, Brazil.; d Diagnósticos da América S.A., São Paulo, Brazil.

**Keywords:** MBTPS2, Ichthyosis congenital, Limb malformations, BRESHECK syndrome, IFAP syndrome, MBTPS2, Ictiose congênita, Malformações dos membros, Síndrome BRESHECK, Síndrome IFAP

## Abstract

**Objective:**

The classic triad, which defines IFAP syndrome, is ichthyosis follicularis, alopecia, and photophobia. It is a rare X-linked genetic disorder characterized by multiple congenital anomalies with variable severity, caused by pathogenic variants in the *MBTPS2* gene, which encodes a zinc metalloprotease that is essential for normal development. This study aimed to report a case of a Brazilian patient with IFAP syndrome presenting skeletal anomalies, which is a rare finding among patients from different families.

**Case description:**

We describe a male proband with IFAP syndrome showing severe ichthyosis congenita, cryptorchidism, limb malformation, and comprising the BRESHECK syndrome features. Using whole-exome sequencing, we identified a rare missense variant in hemizygosity in the *MBTPS2* gene, which had not been identified in other family members.

**Comments:**

This is the first diagnosis of IFAP syndrome in Brazil with a molecular investigation. The present case study thus expands our knowledge on the mutational spectrum of *MBPTS2* associated with IFAP syndrome.

## RESUMO

**Objetivo:**

A clássica tríade de ictiose folicular, alopecia e fotofobia dá nome a uma síndrome rara de origem genética com herança ligada ao cromossomo X (síndrome IFAP, do inglês *Ichthyosis Follicularis*, *Alopecia*, and *Photophobia*). Esta é uma síndrome caracterizada por múltiplas anomalias congênitas de expressividade variável, causada por variantes patogênicas no gene *MBTPS2*, que codifica uma zinco-metaloprotease essencial para o desenvolvimento normal humano. O objetivo deste estudo é apresentar o relato de caso de um paciente brasileiro com síndrome IFAP que apresentou anomalias esqueléticas, um achado raro entre os pacientes de diferentes famílias.

**Descrição do caso:**

Apresentamos um probando do sexo masculino com síndrome IFAP, com ictiose congênita grave, criptorquidia, malformação de membros e as características da síndrome de BRESHECK. Por meio do sequenciamento do exoma completo, identificamos uma variante rara do tipo missense, em hemizigose, no gene *MBTPS2*, não identificada em outros membros da família.

**Comentários:**

Este é o primeiro diagnóstico de síndrome IFAP no Brasil com investigação molecular. A análise molecular e a descrição de uma variante rara no gene *MBPTS2* expandem nosso conhecimento sobre o espectro mutacional desse gene associado à síndrome IFAP.

## INTRODUCTION

The ichthyosis follicularis, alopecia, and photophobia (IFAP) syndrome is a rare X-linked disorder characterized by multiple congenital anomalies with variable severity.^1^ Some patients may also present additional features comprising the BRESHECK/BRESECK syndrome with intellectual disability, brain anomalies, Hirschsprung disease, corneal opacifications, kidney dysplasia, cryptorchidism, cleft palate, and skeletal malformations.^2^ Since its mode of inheritance is X-linked, the IFAP syndrome mostly affects males, which present the full-blown clinical phenotype, while in female carriers, the trait may be nonpenetrant or show minor symptoms.

IFAP syndrome with or without BRESHECK syndrome (MIM#308205) is caused by pathogenic variants in the membrane-bound transcript factor protease site 2 (*MBTPS2*) gene, a zinc metalloprotease essential for cholesterol homeostasis and endoplasmic reticulum stress response.^3^ To date, nearly 60 cases of IFAP syndrome have been reported worldwide. Here, we present an IFAP syndrome case with a rare *de novo* variant in the *MBTPS2* gene.

## CASE REPORT

The male proband is the child of a healthy nonconsanguineous Brazilian couple, a 31-year-old mother and a 33-year-old father, and at the time of his first clinical evaluation, he was 1 month old. The proband has an unaffected brother, and the mother denied any illnesses or drug exposure during the pregnancy that was positive for polyhydramnios and intrauterine growth retardation. He was born from a vaginal delivery at 35 weeks of pregnancy with 1.850 kg (<10th percentile), 38 cm height (<10th percentile), and with a head circumference of 30 cm (<10th percentile). At birth, the patient was noted to have a severe cutaneous abnormality with collodion membrane and congenital ichthyosis with taut skin in the face, eclabium, ectropion, microcephaly, cryptorchidism, flattened rudimentary ears, and absence of hair in the scalp, eyebrows, and eyelashes. He also had upper limb malformations characterized by the absence of the right hand, bilateral shortening of the ulna with radius aplasia, fixed elbows, oligodactyly in the right hand with only the fifth digit present, and in the left foot with the absence of the fourth digit. A cardiac ultrasound revealed a patent foramen ovale, and cholesterol biosynthesis pathway testing results were normal.

The patient and his family were seen at the Pontifical Catholic University of Paraná in Curitiba, Brazil, where samples of peripheral blood were collected for genetic testing. Genomic DNA was extracted from biological material following standard procedures. The proband, his unaffected parents, and his brother were submitted to the Baylor-Hopkins Center for Mendelian Genomics (BHCMG) study through the PhenoDB web-based portal (http://phenodb.net)^4^ for consideration of genetic investigation. The Johns Hopkins Institutional Review Board provided local approval for this study, and informed consent was obtained from the parents, which explicitly allowed publication of photographs of the proband.

The patient was first screened for microscopic and submicroscopic chromosomal alterations using the G-banded karyotype and array-CGH analysis, all with normal results. Whole-exome sequencing from the proband and his unaffected parents and brother (kit SureSelect XT Human All Exon V4 from Agilent Technologies, CA, USA) was performed for the detection of single-nucleotide variants and small insertions and deletions, using the HiSeq2500 platform from Illumina (CA, USA). Targeted regions include the coding DNA and untranslated regions. The medium vertical coverage was 100×, horizontal coverage ≥97.5% at 10× and ≥95% at 20×. The raw paired-end reads were aligned to the GRCh37/hg19 reference genome using the Burrows-Wheeler Aligner. Sequencing data were analyzed using the PhenoDB web-based portal (http://phenodb.net). Variant call format files were used for annotation and filtering of genetic variants. Visual verification of the findings was made using data from the Binary Alignment Map (BAM) files with Integrative Genomics Viewer. The following annotation and filtering strategies were used:

1. Variants with population allelic frequency <1% (1000 Genomes; dbSNP; dbVar);
2. Variants in disease-causing genes (OMIM);
3. Missense and loss-of-function variants; and
4. Variants not located in segmental duplications (excluding pseudogenes).

The detected variants were classified according to the *American College of Medical Genetics and Genomics* (ACMG) guidelines^5^ as pathogenic, likely pathogenic, benign, likely benign, or variants of unknown significance (VUS). We tested all modes of inheritance, starting with a dominant hypothesis (investigation of *de novo* variants), followed by recessive autosomal or X-linked (homozygosity, compound heterozygosis, and hemizygosity). Sanger sequencing was used to validate and perform segregation analysis of the variant in the family.

**Figure 1.**
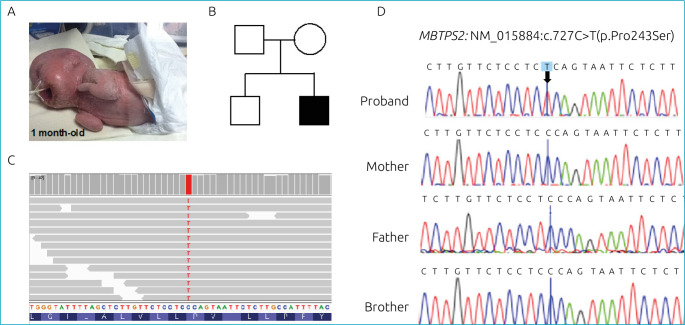
Clinical and genetic data of a patient with ichthyosis follicularis, alopecia, and photophobia (IFAP) syndrome. (A) Image of the 1-month-old patient. (B) Pedigree of the family showing the proband as an isolated case. (C) Image of the BAM file showing the region of the *MBTPS2* variant (NM_015884:exon6:c.C727T:p.P243S) in the proband. (D) Sanger sequencing validated the *MBTPS2* variant in the proband, and segregation analysis of family members determined that the variant segregates only with the affected phenotype.

As a result, we identified a rare *de novo* missense variant (NM_015884:c.727C>T) in hemizygosity in the *MBTPS2* gene. This variant was not present in his parents or brother and has only one entrance on the ClinVar database. Following the ACMG guidelines, the variant was classified as likely pathogenic (PM2/PP3/PS2). Figure 1 presents the case and shows an image of the BAM file displaying the region of the *MBTPS2* variant in the proband. Sanger sequencing validated the variant and confirmed its segregation only with the affected phenotype in the family. We also identified two novel missense variants (NM_000213:c.4217G>C and NM_001005619:c.1940C>T) in heterozygosity in the *ITGB4* gene that could also be associated with the patient’s skin condition. Both variants were also classified as likely pathogenic (PM2/PS2 and PM2/PS2) and were not seen in any of the population databases consulted.

## DISCUSSION

Variants in the *MBTPS2* gene have been reported to cause a broad phenotypic spectrum of X-linked genodermatosis, including the X-linked form of Olmsted syndrome and IFAP syndrome with or without BRESHECK syndrome.^6^ *MBTPS2* maps to chromosome Xp22.12-p22.11 and has 11 exons and encodes 2 isoforms, the longest of which is a 519 amino acid intramembrane zinc metalloprotease, which is essential for development. This metalloprotease functions in the signal protein activation involved in sterol control of transcription and in the endoplasmic reticulum stress response;^7^ it is expressed in the heart, placenta, lung, liver muscle, kidney, and pancreas.^8^ Interestingly, only missense and intronic variants in *MBTPS2* affecting transcription have been described so far. It has been proposed that male embryos do not tolerate total loss of *MBTPS2* and that a residual enzyme activity is required for survival.^9^

The missense variant in *MBTPS2* found in our proband (NM_015884:c.727C>T) has only one entrance on the ClinVar database, such as VUS, but it was described neither in previous studies related to genotype-phenotype^9,10^ nor in other public variant databases such as ExAC and gnomAD. In addition, it is a *de novo* variant in hemizygosity not seen in any family member. The amino acid change occurs in a transmembrane domain, and according to the Revel metapreditor tool, it is predictive of being pathogenic (score 0.7929). In Brazil, there has been only one report of a patient with IFAP syndrome, but without any molecular investigation, the diagnosis was made on the clinical features.^11^

Although the classic triad that defines IFAP syndrome is ichthyosis follicularis, alopecia, and photophobia, the clinical phenotype varies considerably, overlapping with other clinically recognized syndromes. Generalized lamellar desquamation or well-demarcated psoriasiform plaques in addition to follicular hyperkeratosis have been observed. The skin’s histopathology is nonspecific and consists of dilated hair follicles with keratin plugs extending above the surface of the skin. At birth, our patient was noted to have severe congenital ichthyosis. He received systemic retinoid treatment with Acitretin, which significantly improved his skin lesions. Retinoids, such as Acitretin, have been used in genetics disorders of keratinization in children due to its action in the mechanisms of cell proliferation and differentiation. It is relevant to mention that we also identified two novel missense variants in heterozygosity in the *ITGB4* gene, and we cannot exclude the possibility that they can be associated with our patient’s skin condition. *ITGB4* encodes the integrin β4 subunit, which plays a particularly important role in strengthening and stabilizing the skin. It is a component of hemidesmosomes, which are microscopic structures that anchor the outer layer of the skin (the epidermis) to underlying layers. Pathogenic variants in this gene have been associated with other genetic disorders such as epidermolysis bullosa with pyloric atresia, junctional epidermolysis bullosa, and cancer.

Further nonconsistent features related to IFAP syndrome include neurological abnormalities, failure to thrive, nail dystrophy, and renal, vertebral, and testicular abnormalities. In particular, despite the fact that skeletal anomalies are not considered a common feature in this syndrome, our patient presents upper limb malformations – a rare finding among IFAP patients from different families. A review of the literature on *MBPTS2* revealed a previous patient described by Pietrzak et al.^12^ with similar cutaneous and skeletal anomalies, such as aplasia of the ulnar bone and oligodactyly. This patient also carries a variant in *MBTPS2* (c.1001G>A:p.Cys334Tyr).

We describe a male proband with severe ichthyosis and skeletal abnormalities and a rare *de novo MBTPS2* missense variant affecting the transmembrane domain. We suggest that this variant might result in conformational modifications affecting the residual activity and, thereafter, affecting sterol-regulated expression and unfolded protein response. This could lead to possibly undetected functions that might explain the developmental anomalies, such as the skeletal ones presented in our patient.
